# Preparation and Characterization of Novel Polymer-Based Gel Electrolyte for Dye-Sensitized Solar Cells Based on poly(vinylidene fluoride-co-hexafluoropropylene) and poly(acrylonitrile-co-butadiene) or poly(dimethylsiloxane) bis(3-aminopropyl) Copolymers

**DOI:** 10.3390/ma13122721

**Published:** 2020-06-15

**Authors:** Krzysztof Artur Bogdanowicz, Dariusz Augustowski, Justyna Dziedzic, Paweł Kwaśnicki, Wacław Malej, Agnieszka Iwan

**Affiliations:** 1 Military Institute of Engineer Technology, Obornicka 136 Str., 50-961 Wroclaw, Poland; malej@witi.wroc.pl; 2 Research & Development Centre for Photovoltaics, ML System S.A. Zaczernie 190G, 36-062 Zaczernie, Poland; augustowski@doctoral.uj.edu.pl (D.A.); justyna.dziedzic@mlsystem.pl (J.D.); pawelkwasnicki@kul.lublin.pl (P.K.); 3 Department of Advanced Materials Engineering, Faculty of Physics, Astronomy and Applied Computer Science, Jagiellonian University, Łojasiewicza 11, 30-348 Kraków, Poland; 4 Department of Physical Chemistry and Physicochemical Basis of Environmental Engineering, Institute of Environmental Engineering in Stalowa Wola, John Paul II Catholic University of Lublin, Kwiatkowskiego 3A, 37-450 Stalowa Wola, Poland

**Keywords:** dye-sensitized solar cells, gel electrolytes, poly(vinylidene fluoride-co-hexafluoropropylene), poly(acrylonitrile-co-butadiene), poly(dimethylsiloxane) bis(3-aminopropyl)

## Abstract

Polymer gel electrolytes based on poly(vinylidene fluoride-co-hexafluoropropylene) (PVDF-HFP) and poly(acrylonitrile-co-butadiene) (PAB) or poly(dimethylsiloxane) bis(3-aminopropyl)-terminated (PDES-bAP) copolymers were prepared and investigated in dye-sensitized solar cells (DSSCs). Selected optical and electrochemical properties of all compositions with various ratio from 9:1 to 6:4 were investigated towards DSSC applications. The highest value of power conversion efficiency equal to 5.07% was found for DSSCs containing a PVDF-HPF:PAB (9:1) gel electrolyte. Compositions of electrolytes were additionally tested by electrochemical impedance spectroscopy. The influence of the ratio and type of polymers used as an additive to PVDF-HPF on absorption wavelengths, energy gap, and Highest Occupied Molecular Orbital (HOMO) and Lowest Unoccupied Molecular Orbital (LUMO) levels were investigated. Individual components of DSSCs, such as the TiO_2_ layer and platinum nanoparticles, were imaged by scanning electron microscope. Finally, a DSSC module with six electrically separated solar cells with a 7 × 80 mm^2^ active area was constructed based on gel electrolytes and tested.

## 1. Introduction

As a consequence of the rapid rise in the use of electronic devices, the consumption of world power has accelerated as well. This situation involves augmented worldwide electric power production mainly in the form of fossil fuels, which has a tremendous impact on the global environment. To prevent nature from being devastated by, for example, the enormous production of greenhouse gases and air pollution, scientists and engineers around the world are developing numerous generators fueled by renewable energy sources. One of the main ideas to achieve this is to harvest the tremendous amount of energy that comes from the Sun to the Earth’s surface. After many years of research, which were focused on *p–n* junction architectures of the first and the second generation of solar cells, a third generation of photovoltaic devices have emerged to replace previous generations. Originated by Grätzel [1], dye sensitized solar cells (DSSCs) have the potential to become cost-effective, easy to produces and eco-friendly sources of electric energy.

After years of improving efficiency [2,3], durability [4], and production techniques of the DSSCs [5,6], a few problems still need to be addressed. The DSSCs usually contain a dyed mesoporous TiO_2_ layer on the SnO_2_:F conductive transparent glass (FTO), sealed by a lamination foil with a counter electrode which is composed of platinum nanoparticles deposited on a second FTO glass. The electrical contact is enabled by the presence of a liquid electrolyte between them. The main disadvantages of commercially used liquid-based electrolytes are:

- A tightness problem, namely, leakage or volatilization from the devices caused by high vapor pressure;
- The chemical components of the electrolyte lead to corrosion of the platinum counter electrode;
- Low thermal resistance; freezing can cause physical damage [2,3,4,7].

The solution to this complication could be the implementation of solid-state [8] and quasi-solid-state electrolytes [9,10,11]. However, the first one exhibits poor contact with TiO_2_, which causes low solar-to-electric power conversion efficiency (PCE). Therefore, quasi-solid-state electrolytes provide better filling and contact with the mesoporous structure of a working electrode, and it seems to be the best solution. Composed of polymers, gel-type electrolytes possess high ionic conductivity, trapping solvent molecules in cages formed by the polymer’s three-dimensional matrix [12].

In the literature different polymeric systems for DSSCs, such as poly(vinylidene fluoride-co-hexafluoropropylene) (PVDF-HFP) with poly (acrylonitrile-co-vinyl acetate) (PAN-VA) [11,13,14,15,16,17,18,19] or electrolytes based on PVDF-HFP and liquid electrolytes, containing imidazolinium ionic liquids of different carbon chain lengths [20] were tested. Since fluorine has a smaller ionic radius and high electro negativity, PVDF-HFP is known to enhance the ionic conductivity [13] and is widely investigated as a main component of gel electrolytes. Moreover, compositions, such as poly(ethylene oxide)-poly-(vinylidene fluoride) [21], poly(acrylonitrile) [15], poly(methyl acrylate) [22], or poly(methyl methacrylate) [4], were prepared and tested in quasi-solid state DSSCs.

Among other polymers used as a component of solid electrolytes, poly(acrylonitrile-co-butadiene) (PAB) was reported in the literature [23,24,25]. Matsumoto [23] used poly(acrylonitrile-co-butadiene) rubber (NBR) and poly(styrene-co-butadiene) rubber (SBR) in order to form a dual phase solid structure able to convey cations through membrane structure. The PAB, doped with lithium salt solution, formed conductive channels achieving a conductivity of 7.2 × 10^4^ S cm^−1^ for a system containing 1 M LiClO_4_ in 50/50 (v/v) γ-butyrolactone (γ-BL)/ 1,2-dimethoxyethane (DME) solution [23]. Also, Chae at al. [24] used a PAB polymer as a dopant to poly(pyrrole) in order to increase the conductivity of the whole system. Despite the fact that PAB copolymer, as a polymeric electrolyte, is able to form coordination bonds with cations, such as Li^+^ (only through nitrile side groups), because the C=C present in butadiene groups of are inert with respect to Li^+^ [25], it was never used as a component of a DSSC electrolyte.

Another polymer studied as component of polymeric electrolytes was poly(dimethylsiloxane) (PDMS or PDES in this work) [26,27]. This polymeric family possesses the ability to cause phase segregation of polymeric gel morphologies, enhancing the cation conductivity. Moreover, Li et al. [27] managed to prepare a PVDF/PDMS blended separator, using a mass ratio of 7/3, respectively; ionic conductivity was found to be 1.17 × 10^−3^ S cm^−1^ after liquid electrolyte uptake reached 250 wt%. Thanks to the specific porous structure of the membrane, the electrolyte uptake may have been the critical factor that was responsible for the increase of ion conductivity, compared to PVDF neat membrane [27].

Here, we report an efficient organic polymer-based electrolyte prepared by poly(vinylidene fluoride-co-hexafluoropropylene) (PVDF-HFP) doped, to the best of our knowledge for the first time, with poly(acrylonitrile-co-butadiene) (PAB) or poly(dimethylsiloxane) bis(3-aminopropyl)-terminated (PDES-bAP) copolymers. The novel compositions of electrolyte were studied to obtain comprehensive information about its selected physicochemical properties. Furthermore, 7 × 7 mm^2^ DSSC devices were prepared and characterized by current–voltage characteristics and scanning electron microscopy. Finally, a DSSC module with six solar cells with a 7 × 80 mm^2^ area containing gel electrolytes developed by us was constructed based on these gel electrolytes and tested. In this work, we studied the influence of a new polymer composition on the gelling properties of electrolyte, its electrical properties, and the scaling-up of the single-cell device to a large area module system.

## 2. Experimental

### 2.1. Materials

All chemical components and substrates were bought from a marketable source. For example, the FTO (SnO_2_:F) glass (NSG TEC^TM^ A7, 6–8 Ω/square) substrate was purchased from Pilkington (Sandomierz, Poland). Titania paste (18NR-T), platinum paste (PT1), and di-tetrabutylammonium cis-bis(isothiocyanato)bis(2,2’-bipyridyl-4,4’dicarboxylato)ruthenium(II) (N719 dye) were purchased from GreatCell Solar (Elanora, Australia). Lamination foil was purchased from DuPont Surlyn® (Wilmington, DE, USA). Poly(vinylidene fluoride-co-hexafluoropropylene) (PVDF-HFP) containing approximately 12 wt.% of hexafluoropropylene and poly(acrylonitrile-co-butadiene) (PAB), containing approximately 37–39 wt.% of acrylonitrile were purchased from Sigma–Aldrich (St. Louis, MI, USA). Poly(dimethylsiloxane) bis(3-aminopropyl) terminated (PDES-bAP), cSt approximately 50–60 was purchased from fluoroChem (Hadfield, UK). Acetonitryl (purity ≥ 99.9%, Honeywell, Charlotte, NC, USA), iodine (purity 99.8%, Chempur, Piekary Śląskie, Poland), and lithium iodine (99.8%, un-hydrated, Merck, Darmstadt, Germany) were used as received.

### 2.2. Preparation of Gel Electrolyte

In order to prepare the gel electrolyte, 10 wt.% of various polymers were dissolved separately in 20 mL of acetonitrile. The process was started by heating the entire mixture to 60 °C for 30 min at a constant stirring speed of 300 rpm. Then, the temperature was raised by approximately 5 °C until reaching a temperature of 80 °C [13]. The dissolution process was carried out until a homogeneous mixture was obtained. The gel electrolyte compositions were obtained by mixing at suitable volume ratios for different polymer solutions. Then, lithium iodide and crystalline iodine were added to achieve concentrations of 0.8 mol/dm^3^ and 0.08 mol/dm^3^, respectively. Table 1 presents the compositions of gel electrolytes. The final gel electrolyte composition is included with its composition and whole solvent used in the preparation process. The loss of solvent was, in some cases, at the most 0.5 mL, which was at the most 4.5 wt.% of the whole composition. The amount of unbonded solvent was removed, and its volume was measured only in the case of the gelling process in vials. The electrolyte was prepared in a sealed flask to avoid major loss of solvent during the preparation process. The advantage in using a gel electrolyte in the liquid form facilitated the cell filling process, especially in the case of large area cells.

**Figure 1:**
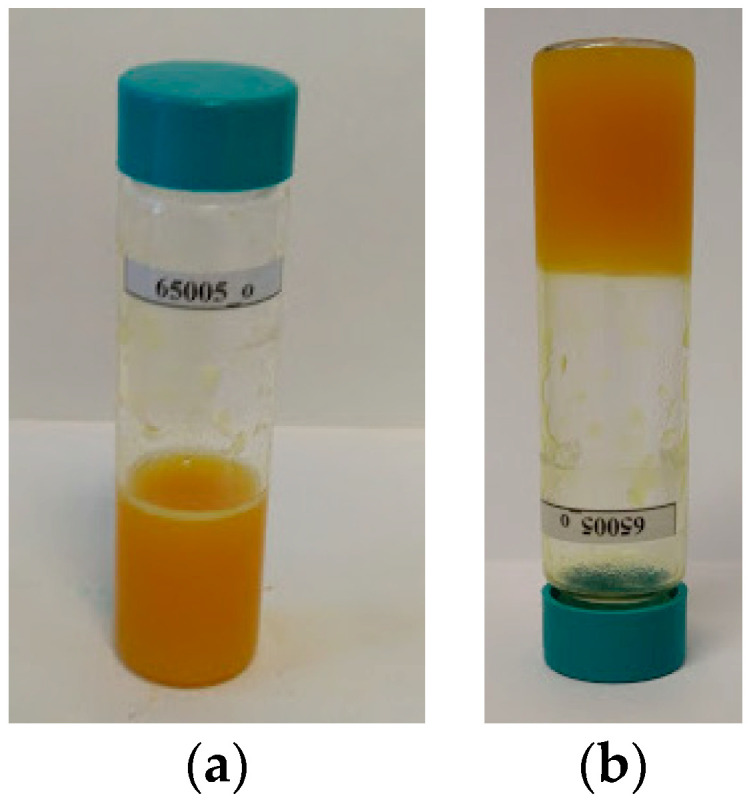
Gel electrolyte based on poly(vinylidene fluoride-co-hexafluoropropylene) and poly(dimethylsiloxane) bis(3-aminopropyl) terminated (PVDF-HFP:PDES-bAP) (9:1) before (**a**) and after (**b**) the gelation process.

In the initial stage, after mixing the two polymeric solutions, the samples were kept at a constant temperature of 80 °C. Then, the temperature was lowered to 65 °C. The decrease in temperature did not affect fluidity and homogeneity, enabling sampling for analysis. The gelation process was carried in hermetically sealed glass bottles and, for all samples, occurred slowly at room temperature up to 10 days. In order to accelerate the gelation, the process was carried out for all samples by placing freshly prepared gel electrolyte compositions by placing them for 60 min at −22 °C and then stored at room temperature (20–25 °C). Figure 1 presents the full gel electrolyte freshly prepared and after the gelling process.

### 2.3. Preparation of DSSC Devices

The working electrodes in the DSSC devices were prepared on a SnO_2_:F glass substrate (FTO). The substrates were cleaned in an ultrasonic bath ultrasonic bath (EMAG, Emmi-20 HC, Mörfelden-Walldorf, Germany) with acetone and isopropanol sequentially for 15 min and then dried under nitrogen flow. A mesoporous TiO_2_ layer was deposited via a screen-printing method from titania paste. Afterwards, the electrode was annealed at 565 °C. The thickness of the TiO_2_ layer was found to be 11 μm using a stylus profilometer. In the dye-sensitization process, 10^−4^ mol/dm^3^ ethanol solution of N719 dye was used and the process lasted 24 h.

To obtain the uniform doping of dye molecules to TiO_2_ nanoparticles, the vessel with dye solution was placed on the orbital shaker. A low rotational speed of 50 rpms provided a stable stirring of the solution during the sensitization process. The counter electrode was also prepared by a screen-printing method using platinum paste on FTO glass and then annealing at 565 °C.

Both electrodes were sealed with 60 μm lamination foil. Devices were filled with previously prepared gel electrolyte after one hour of water bath at 80 °C to receive a homogeneous solution. The same preparation process and materials were employed to prepare 7 × 7 mm^2^ and 7 × 80 mm^2^ solar cells devices. Only different sizes of screens were used to receive specific active area dimensions during screen printing. In the case of the DSSC module, the electrical separation was achieved by laser ablation of the conducting layer (DK Lasertechnik, λ = 1064 nm, Cracow, Poland), and, thus, forming six strips of disjointed FTO areas.

### 2.4. Characterization

The transmission Ultraviolet–Visible (UV-Vis) spectra were acquired using an A360 UV-Vis spectrophotometer (AOR Instruments, Shanghai, China) with an interval of 0.2 nm and medium scan speed. Prior to the study, samples of electrolyte containing only the polymeric components were previously spin-coated on glass supports at 500 rpm for 20 s.

Electrochemical measurements were carried out as described in our previous work [28]: Metrohm Autolab PGSTAT M204 potentiostat (Metrohm AG, Barendrecht, Nederland) and the electrochemical cell contained a glassy carbon electrode (diameter = 2 mm), a platinum rod, and Ag/AgCl as working electrode, counter and reference electrodes were used, respectively. Potentials are referenced with respect to ferrocene (Fc) which was used as the internal standard. Cyclic voltammetry experiments were conducted in a standard one-compartment cell, in acetonitrile (Honeywell, Charlotte, N.C., USA, ≥ 99.9%), under argon. 0.2 M Bu_4_NPF_6_ (Alfa Aesar, Haverhill, MA, USA, 99%) was used as the supporting electrolyte. The sample was prepared by diluting 1 mL of the original polymer solution by adding 9 ml of acetonitrile with supporting electrolyte. The deaeration of the solution was achieved by argon bubbling through the solution for about 15 min prior to the measurement. All electrochemical experiments were carried out at ambient temperature and pressure.

Electrochemical impedance spectroscopy was performed using Metrohm Autolab PGSTAT M204 potentiostat in potentiostatic mode. The amplitude of 10 mV and 71 different frequencies ranging from 100 kHz to 0.1 Hz were selected. Current ranging was set automatically from 100 mA to 10 nA. The experimental setup was built of a glass cell with two silver wires as electrodes and a volume of tested electrolytes equal 0.15 cm^3^. A distance of 1 cm between electrodes was set for all measurements.

The thickness of the TiO_2_ layers were measured by a stylus profilometer (Bruker Dektak XT, Billerica, MA, USA). 

A Scanning Electron Microscope Quanta™ 3D FEG (FEI, Hillsboro, OR, USA) was used to determine the individual components of the DSSCs. 

The performances of the DSSC devices were investigated using a solar simulator (CLASS-01, PV Test Solutions, Wrocław, Poland) under AM1.5 illumination with 100 mW/cm^2^ light intensity.

## 3. Results and Discussion

### 3.1. Effect of Gel Compositions Based on PVDF-HFP and PAB or PDES-bAP on the Optical and Electrochemical Properties

**Scheme 1:**
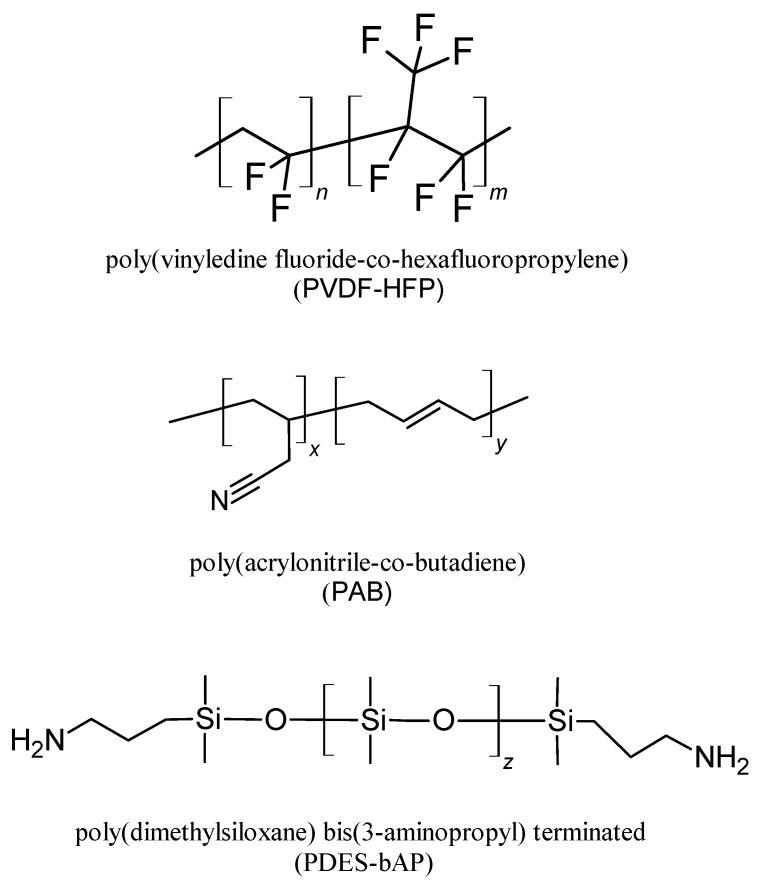
Chemical structure of polymers used as components of gel electrolytes.

In this work, we investigated eight polymer compositions based on poly(vinylidene fluoride-co-hexafluoropropylene) (PVDF-HFP) and poly(acrylonitrile-co-butadiene) (PAB) or poly(dimethylsiloxane) bis(3-aminopropyl)-terminated (PDES-bAP) copolymers (see Table 1) towards application in DSSCs. The chemical structures of polymers used as components of gel electrolytes are presented in Scheme 1.

It is important that the polymeric components of gel electrolytes should possess the absorbance out of the range of dye used in DSSCs. We investigated the absorption spectra of polymeric components of gel electrolytes spun-casted on a glass substrate. The layers were made with a spin-coater (500 rpms, 20 s) on glass in order to reduce the intensity of polymers enabling spectra collection for absorbances below 1.

Table 2 presents the value of the energy gap, based on λ_offset_, and the maximum absorption band of the investigated polymer compositions. 

**Figure 2:**
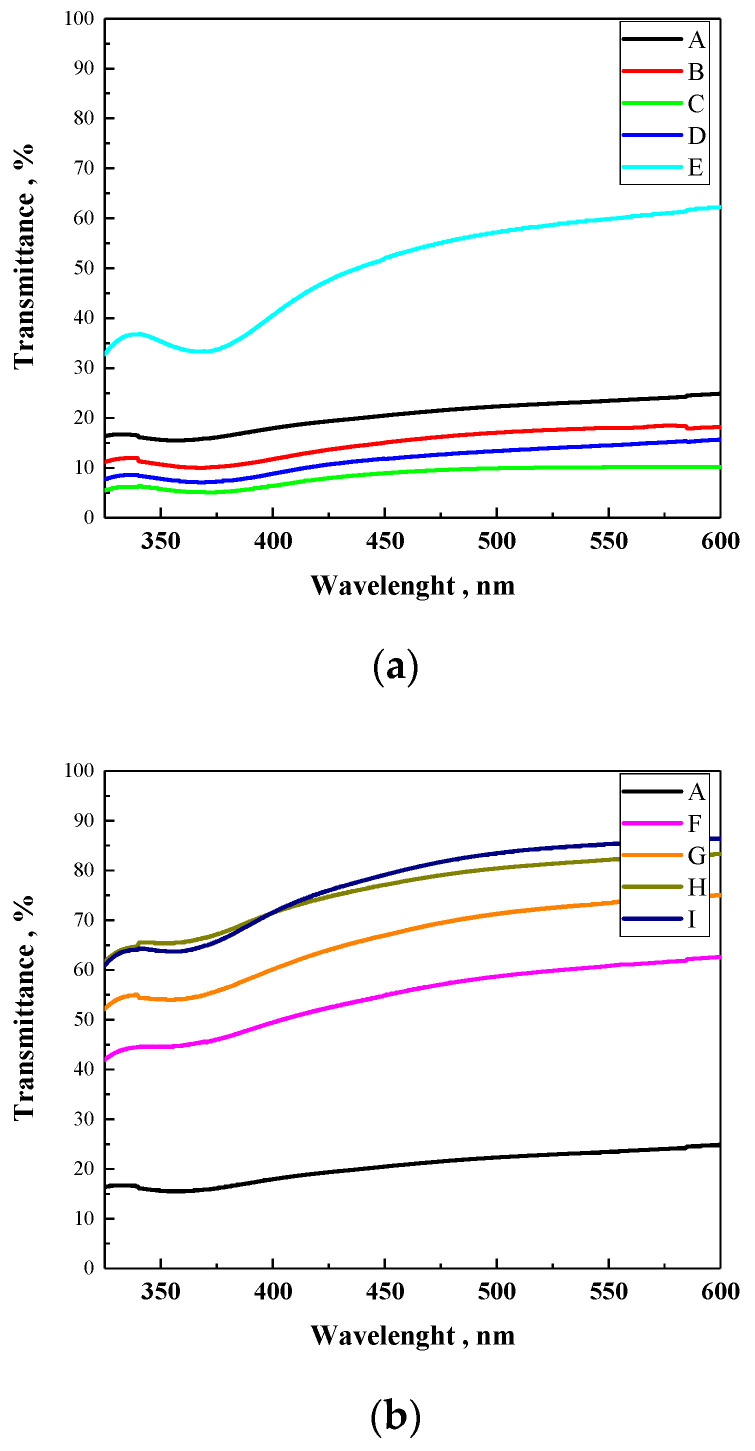
UV-Vis transmittance spectra in film for all investigated electrolytes, where (**A**) PVDF-HFP; (**B**) PVDF-HFP:PAB (9:1); (**C**) PVDF-HFP:PAB (8:2); (**D**) PVDF-HFP:PAB (7:3); (**E**) PVDF-HFP:PAB (6:4); (**F**) PVDF-HFP:PDES-bAP (9:1); (**G**) PVDF-HFP: PDES-bAP (8:2); (**H**) PVDF-HFP: PDES-bAP (7:3); (**I**) PVDF-HFP: PDES-bAP (6:4), (**a**): A, B, C, D, E and (**b**): A, F, G, H, I.

In all cases, only a single band was observed centered at: 358 nm for PVDF-HFP; 370 nm for all PVDF-HFP:PAB mixtures; and 362 nm for all mixtures of PVDF-HFP:PDES-bAP. Absorbance of all polymeric compositions was out of the absorption range reported in the literature for the N719 dye (e.g., 370 nm and 500 nm) [29]. Moreover, the intensity of the thin layer suggests that the presence of polymers should not affect efficiency of a DSSC due to its translucidity. Additionally, Figure 2 presents collective UV-Vis transmittance spectra for all investigated compositions. In the range from 400 nm to 600 nm, PVDF-HFP:PDES-bAP compositions showed higher transmittance than PVDF-HFP:PAB compositions (see Figure 2).

The energy gap (E_g_^opt.^) was estimated from the equation: E_g_^opt^ = 1240/λ_offset_, where λ_offset_ is the wavelength (in nm) of the absorption offset [30], and their values ranged from 2.44 eV for PVDF-HFP to 2.88 eV for PVDF-HPF:PDES-bAP (7:3). Taking into consideration the ratio of second polymer added to PVDF-HFP, we observed for ratio 9:1 and 8:2 for PVDF-HPF:PAB compositions which increased the value of E_g_ by approximately 0.26–0.28 eV; while for ratio 7:3 E_g_, it increase by approximately 0.2 eV compared with PVDF-HPF. For PVDF-HPF:PDES-bAP compositions, the lowest value of E_g_ compared with all investigated compositions was found for ratio 9:1. No clear trend was observed between the polymer composition and calculated E_g_, which might be caused by the error related to assessing λ_offset_—a very broad signal.

As a following step, we evaluated the electrochemical properties of the polymeric components of the gel electrolyte. It has been reported in the literature [26,27] that a mixture of two polymers, especially with different polarities, causes phase separation; this is particularly observed in cases of solid structures. In cases of polymeric gels, due to the gelation process and diffusion (taking place between liquid phases formed by polymer-rich and polymer-poor phase) separation could occur. Another factor that needs to be taken into account is the increase in viscosity during gelation, which slows down the phase separation process [31]. In our case, we are dealing with a much more complex system; the gel electrolytes were composed of two polymers, in different ratios, present in acetonitrile which could possibly cause several different phase combinations that could affect the results obtained in voltammograms. In order to select the signals corresponding to oxidation reduction signals, we performed consecutive measurements for all gel compositions and pure polymers, and we selected signals that displayed the smallest change in intensity over consecutive measurements. During the entire time, no visible phase separation of gel electrolyte over time was observed.

**Figure 3:**
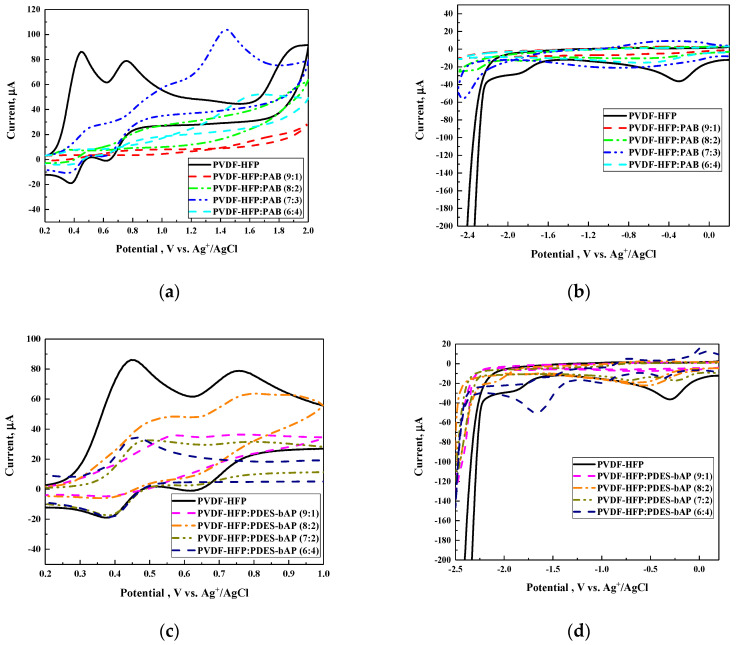

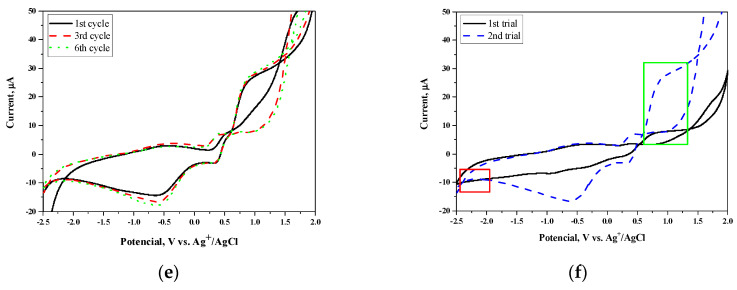
Comparison of voltammograms (oxidation (**a,c**), reduction (**b,d**) for PVDF-HFP and compositions of PVDF-HPF:PAB (**a,b**), PVDF-HPF:PDES-bAP (**c,d**) with different weight ratios and voltammograms of PVDF-HFP:PAB (9:1) for different cycles (**e**) and two trials (**f**)

The experimental values for all HOMO, LUMO, and energy band gaps are displayed in Table 3, and voltammograms are in Figure 3. The order to compare the HOMO energy values obtained from experimental voltammograms are set in a decreasing order of value as follows: PVDF-HPF:PAB (9:1) > PVDF-HPF:PDES-bAP (8:2) > PVDF-HPF:PDES-bAP (6:4) > PVDF-HPF:PDES-bAP (9:1) > PVDF-HPF:PAB (7:3) > PVDF-HPF:PAB (6:4) = PVDF-HPF:PDES-bAP (7:3) > PVDF-HPF:PAB (8:2) > PVDF-HPF according to their values −2.16 eV, −2.20 eV, −2.21 eV, −2.29 eV, −2.32 eV, −2.33 eV, −2.39 eV and −2.45 eV, respectively. All two component polymer compositions showed HOMO energy lower than the main component, PVDF-HPF; however, no evident tendency was observed.

Also comparing the LUMO energy values, an array can also be set in decreasing order: PVDF-HPF:PDES-bAP (8:2) > PVDF-HPF:PDES-bAP (9:1) > PVDF-HPF:PAB (6:4) > PVDF-HPF:PAB (9:1) > PVDF-HPF:PAB (7:3) > PVDF-HPF > PVDF-HPF:PDES-bAP (7:3) > PVDF-HPF:PDES-bAP (6:4) > PVDF-HPF:PAB (8:2), in accordance to their values −5.14 eV, −5.59 eV, −5.60 eV, −5.71 eV, −6.13 eV, −6.16 eV, −6.17 eV, −6.20 eV, respectively. The lowest LUMO energy value was found for PVDF-HPF:PAB (8:2). Also, in this case, no obvious trend was observed considering composition of samples.

The values of energy gap displayed no tendency regarding relation between its composition and E_g_ value. The arrangement of samples in increasing energy value gives following series: PVDF-HPF:PDES-bAP (9:1) < PVDF-HPF:PDES-bAP (8:2) < PVDF-HPF:PAB (6:4) < PVDF-HPF:PAB (7:3) < PVDF-HPF:PAB (9:1) < PVDF-HPF < PVDF-HPF:PAB (8:2) < PVDF-HPF:PDES-bAP (7:3) < PVDF-HPF:PDES-bAP (6:4), in range from 2.42 eV to 3.96 eV. The difference among energies for different weight compositions can be related to the specific equilibrium between among components in electrolyte mixtures similarly to the results reported in the literature for mixtures of PVDF:PMDS [30].

Additionally, a cyclic voltammetry (CV) experiment for each sample included six cycle measurements in order to establish the stability of the system (Figure 3e). Only small changes were observed; however, the main signals used for HOMO and LUMO energy calculations were not affected. Moreover, to exclude the influence of impurities, we performed repetitive measurement that confirmed that the registered data were not affected by any impurities from the measuring system (Figure 3f).

The E_g_ values obtained from the two different methods (i.e., UV-Vis and CV) were compared, and the results indicate that these values from the CV are generally higher than obtained by UV-Vis studies (see Table 2 and Table 3). It should be stressed that the energy differences between these methods are typically not identical, and differences as large as 1 eV have been reported [30,32,33]. Additional studies to clarify this issue are necessary.

**Figure 4:**
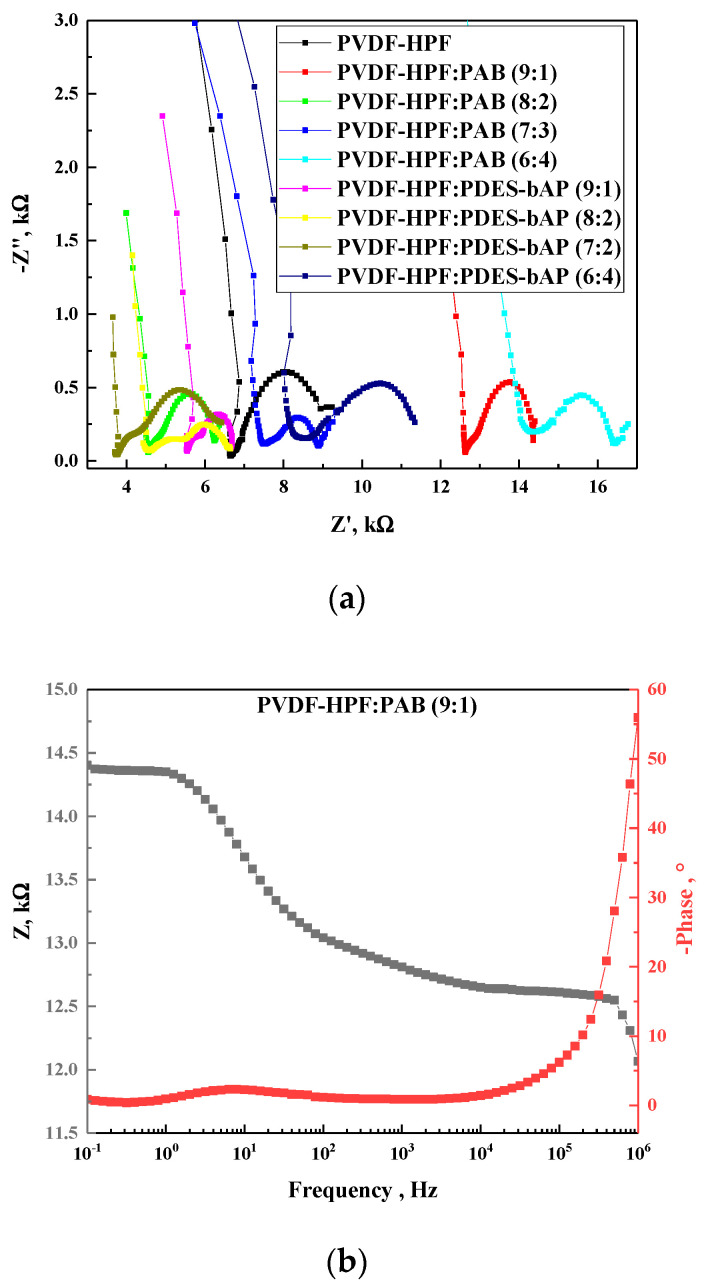
Nyquist plots for all investigated gel electrolytes (**a**) together with Bode plots for gel electrolyte based on PVDF-HFP:PAB (9:1) (**b**).

In order to assess the conductivity of gel electrolyte based on mixtures of polymers PVDF-HFP and PAB or PDES-bAP in different proportions and incorporating lithium iodine and iodine, electrochemical impedance spectroscopy experiments were performed. The Nyquist plot (Figure 4) obtained by the impedance measurement was used to fit the plot of an equivalent circuit build of resistor (R1) connected in parallel to constant phase element (CPE1) and in series to resistor 2 (R2). The first resistance was assigned to charge-transfer at counter electrode (R1), whereas the second resistance (R2) and capacitance (CPE1) represents the diffusion of I^−^/I_3_^−^ in the polymeric gel matrix. It was noticed that the lowest resistance (see Table 4) values were observed for compositions 8:2 and 7:3 both series PVDF-HFP:PAB and PVDF-HFP:PDES-bAP, compared to pure PVDF-HFP.

**Figure 5:**
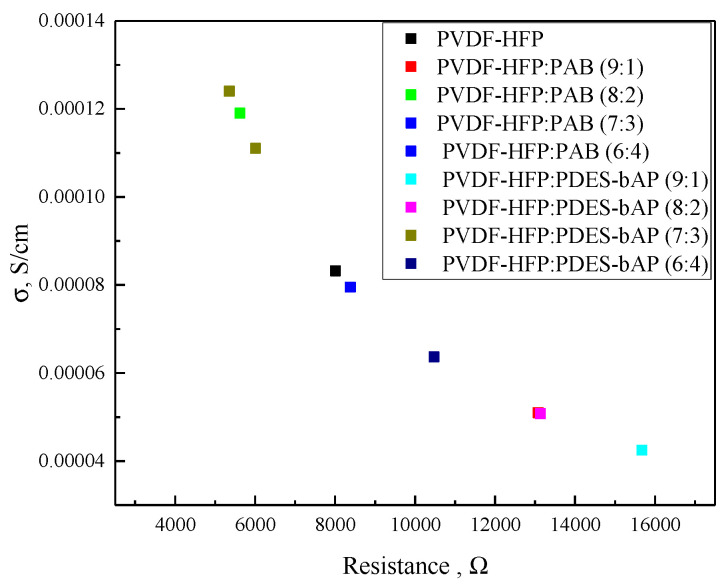
Relationship between resistance and the conductivity of gel electrolytes.

All studied polymer electrolytes exhibit semicircles in Nyquist plots. Moreover, a small drop in values of the real part (Z’) and the imaginary part (Z”) of a polymer composition’s impedance along with a change in the ratio of PVDF-HFP to other polymers can be seen (see Figure 4). Figure 5 represents the relationship between resistance and conductivity, and it can be noticed that the curve demonstrates an almost logarithmic dependence.

### 3.2. Characterization of DSSC Devices

**Figure 6:**
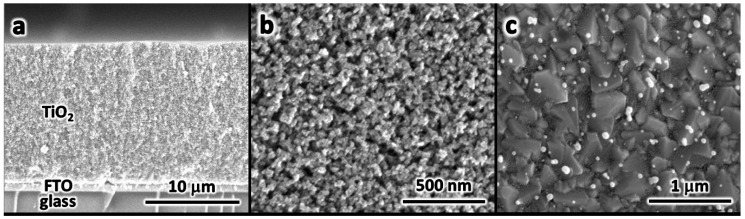
SEM images of (**a**) a photoanode cross-section, (**b**) sintered nanoparticles forming a TiO_2_ mesoporous layer, and (**c**) platinum nanoparticles deposited on FTO counter electrode.

Individual components of a DSSC were imaged using SEM. Figure 6a shows a cross-section of the photoanode (working electrode). As it can be seen in the image glass, FTO thin film and mesoporous TiO_2_ layers are indicated. The image presented as Figure 6b shows a 20 nm mesoporous structure of titanium oxide nanoparticles, formed by sintering at a temperature of 565 °C. A significant size dispersion from 5 to 50 nm of platinum nanoparticles (bright spots) deposited on a counter electrode was observed (Figure 6c). The high temperature during the annealing process may possibly be the reason of the formed Pt agglomerates presented in the FTO Surface.

**Figure 7:**
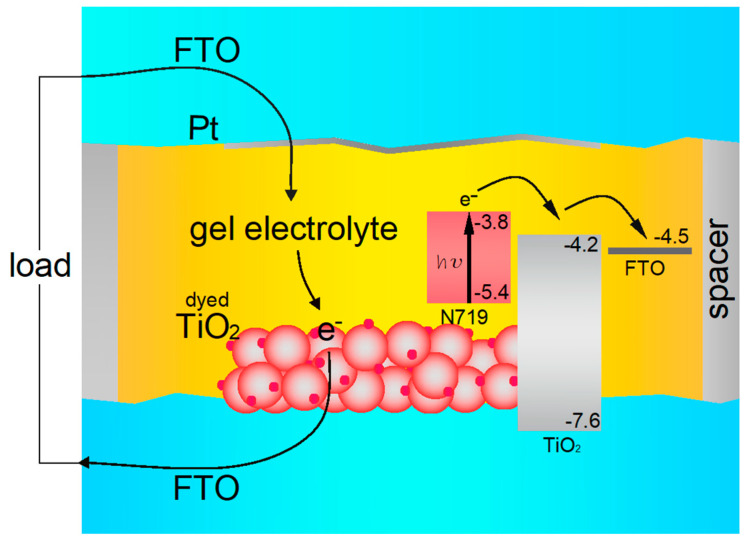
Graphical representation of the working principles for a DSSC cell filled with gel electrolyte.

A graphical representation of working principles for a DSSC cell filled with gel electrolyte is presented in Figure 7.

**Figure 8:**
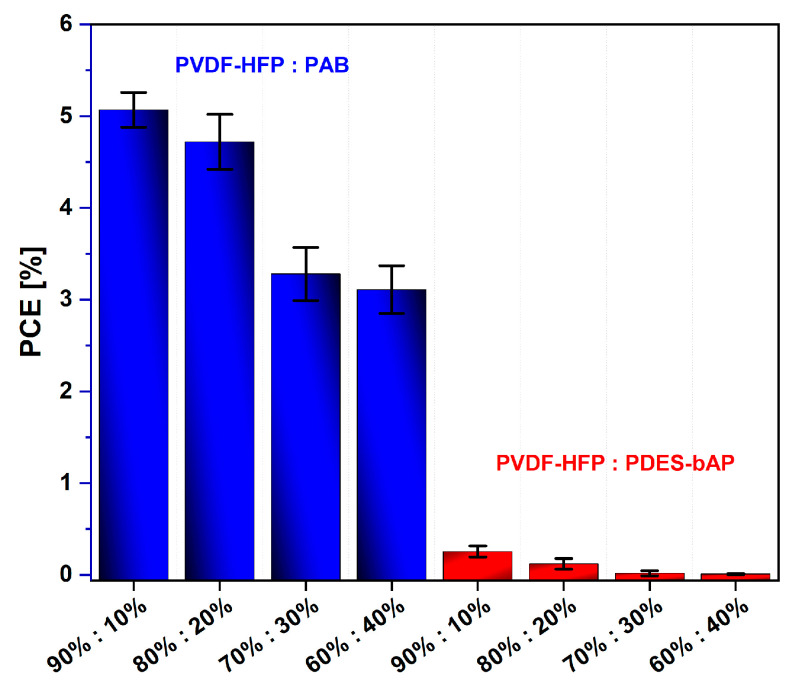
Dependence of PCE from the polymer ratio.

The performance of DSSC devices with all prepared polymer electrolytes were investigated using solar simulator under AM1.5 illumination with 100 mW/cm^2^ light intensity. The best efficiency was obtained for PVDF-HPF:PAB (9:1). For other constructed DSSC devices, values of PCE were found in the range 0.01–5.07% (Figure 8). The devices based on PVDF-HPF:PDES-bAP exhibited values of PCE below 1%. Photovoltaic parameters of all created DSSC based on PVDF-HPF are collected in Table 5.

**Figure 9:**
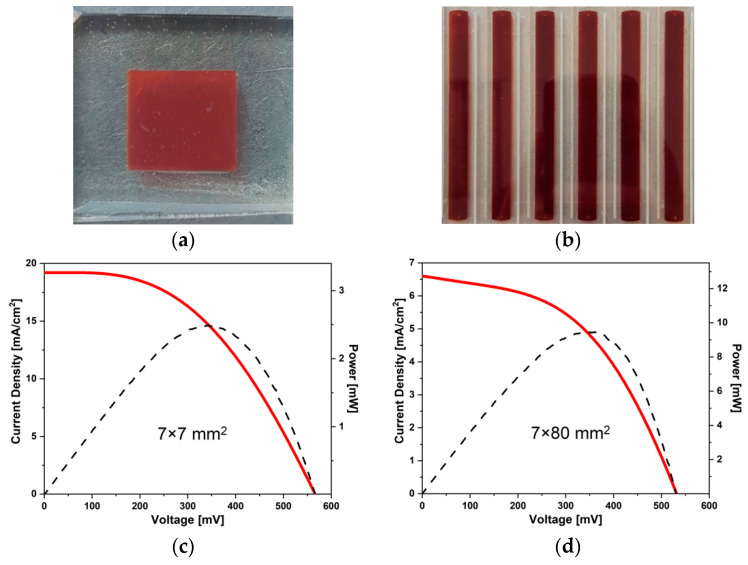
Current-voltage characteristic (red solid) and power curve (black, dashed) of 7 × 7 mm^2^ (**c**) and 7 × 80 mm^2^ (**d**) gel electrolyte dye-sensitized solar cell along with a photo of a small device (**a**) and a module (**b**) we created.

High value of short circuit current was reported to be J_sc_ = 19.31 mA/cm^2^ and open circuit voltage V_oc_ = 567 mV. Device power conversion efficiency (PCE) was equal 5.07% and calculated power (P_max_) equal 2.48 mW (5.06 mW/cm^2^) for 7 × 7 mm^2^ active area. The low value of fill factor FF = 0.47 was caused by high series resistance (R_s_) and equal to 24.7 Ω which caused flattening of current–voltage (V–J) characteristic (shown in Figure 9). For created DSSC module photovoltaic parameters. such as J_sc_, R_s_, R_sh_ and PCE, decreased as an effect of upscaling size area of investigated devices (see Table 5). Worth mentioning is the fact that the value of FF did not change along with the increase in the size of the area of DSSC to 7 × 80 mm^2^.

**Figure 10:**
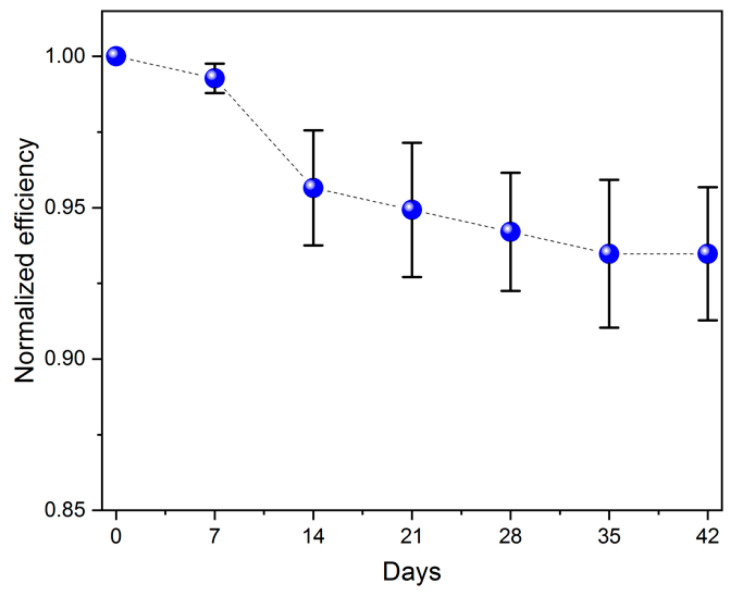
Normalized efficiency of the DSSC module as a function of storage time at ambient conditions.

The long-term performance stability of gel electrolyte was investigated by measuring the efficiency over a one-week interval. The temperature during the measurements was stable (22 ± 1 °C), and the samples were stored at normal conditions. As shown in Figure 10, the parameters were obtained individually from six solar cells in the DSSC module and then averaged. The presented data suggest that the power conversion efficiency dropped significantly after approximately 10 days. Then, after two weeks, the performance of the photovoltaic devices began to plateau. Over a period of 42 days, the average efficiency decreased by 7% (to 1.47%) compared to the initial value of 1.57%.

In this work, eight gel polymer electrolytes compositions were prepared by mixing poly(vinylidene fluoride-co-hexafluoropropylene) (PVDF-HFP) and poly(acrylonitrile-co-butadiene) (PAB) or poly(dimethylsiloxane) bis(3-aminopropyl)-terminated (PDES-bAP) copolymers towards DSSC applications. All investigated compositions exhibited gel properties. We found that depending on the type of second polymeric component in the gel electrolyte based on PVDF-HFP, a significant decrease in the PCE value was also observed. By using the PVDF-HPF:PAB (9:1) composition, the gel DSSC can achieve the highest PCE of 5.07%. Along with an increase in the area of the investigated DSSC, a decreased in the value of PCE was observed. Our study showed that the investigated polymer electrolytes exhibited similar optical and electrochemical properties, but they showed different photovoltaic parameters in the constructed of a DSSC. This behavior can probably be explained by the percolation threshold among the investigated polymers and needs to be further exploration through experimental studies.

## Figures and Tables

**Table 1:** Polymer content in gel electrolytes in relation to the weight of the solvent.

Code	wt.% of PVDF-HFP	wt.% of PAB	wt.% of PDES-bAP
PVDF-HPF	10	-	-
PVDF-HPF:PAB (9:1)	9	1	-
PVDF-HPF:PAB (8:2)	8	2	-
PVDF-HPF:PAB (7:3)	7	3	-
PVDF-HPF:PAB (6:4)	6	4	-
PVDF-HPF:PDES-bAP (9:1)	9	-	1
PVDF-HPF:PDES-bAP (8:2)	8	-	2
PVDF-HPF:PDES-bAP (7:3)	7	-	3
PVDF-HPF:PDES-bAP (6:4)	6	-	4

**Table 2:** Energy gap (E_g_^opt.^) calculated from UV-Vis spectra of investigated polymer compositions.

Code	λ_max_ (nm)	λ_offset_ (nm)	E_g_^opt^ (eV)
PVDF-HPF	358	508	2.44
PVDF-HPF:PAB (9:1)	370	456	2.72
PVDF-HPF:PAB (8:2)	370	460	2.70
PVDF-HPF:PAB (7:3)	370	470	2.64
PVDF-HPF:PAB (6:4)	370	454	2.73
PVDF-HPF:PDES-bAP (9:1)	362	464	2.67
PVDF-HPF:PDES-bAP (8:2)	362	462	2.69
PVDF-HPF:PDES-bAP (7:3)	362	431	2.88
PVDF-HPF:PDES-bAP (6:4)	362	446	2.78

**Table 3:** Values of HOMO, LUMO, and E_g_ values calculated from cyclic voltammetry experiments (CV).

Code	E_ox_^onset^ (V)	E_red_^offset^ (V)	E_HOMO_ (eV)	E_LUMO_ (eV)	E_g_ (eV)
PVDF-HFP	1.68	−2.12	−6.13	−2.45	3.68
PVDF-HFP:PAB (9:1)	1.15	−2.41	−5.60	−2.16	3.44
PVDF-HFP:PAB (8:2)	1.75	−2.18	−6.20	−2.39	3.81
PVDF-HFP:PAB (7:3)	1.26	−2.25	−5.71	−2.32	3.39
PVDF-HFP:PAB (6:4)	1.14	−2.24	−5.59	−2.33	3.26
PVDF-HFP:PDES-bAP (9:1)	0.26	−2.28	−4.71	−2.29	2.42
PVDF-HFP:PDES-bAP (8:2)	0.69	−2.37	−5.14	−2.20	2.94
PVDF-HFP:PDES-bAP (7:3)	1.71	−2.24	−6.16	−2.33	3.83
PVDF-HFP:PDES-bAP (6:4)	1.72	−2.36	−6.17	−2.21	3.96

**Table 4:** Calculated values of resistance and conductance for gel electrolytes.

Code	R (Ω)	L (cm)	A (cm^2^)	σ (S/cm)
PVDF-HFP	8010	1	1.5	8.32 × 10^−5^
PVDF-HFP:PAB (9:1)	13078	1	1.5	5.10 × 10^−5^
PVDF-HFP:PAB (8:2)	5617	1	1.5	1.19 × 10^−4^
PVDF-HFP:PAB (7:3)	8383	1	1.5	7.95 ×·10^−5^
PVDF-HFP:PAB (6:4)	15670	1	1.5	4.25 × 10^−5^
PVDF-HFP:PDES-bAP (9:1)	13132	1	1.5	5.08 × 10^−5^
PVDF-HFP:PDES-bAP (8:2)	6008	1	1.5	1.11 × 10^−4^
PVDF-HFP:PDES-bAP (7:3)	5355	1	1.5	1.24 × 10^−4^
PVDF-HFP:PDES-bAP (6:4)	10473	1	1.5	6.37 × 10^−5^

**Table 5:** Gel electrolyte dye-sensitized solar cell parameters (7 × 7 mm^2^) and (7 × 80 mm^2^).

Code	V_oc_ (mV)	J_sc_ (mA/cm^2^)	P_max_ (mW)	R_s_ (Ω)	R_sh_ (kΩ)	FF (-)	PCE (%)
**For size of DSSC 7 × 7 mm^2^**							
**PVDF-HFP:PAB (9:1)**	567 ± 6.4	19.31 ± 0.61	2.48 ± 0.18	24.7 ± 2.0	1.21 ± 0.34	0.47 ± 0.01	5.07 ± 0.19
**PVDF-HFP:PAB (8:2)**	567 ± 9.0	18.53 ± 0.69	2.32 ± 0.14	56.6 ± 6.3	1.31 ± 0.63	0.45 ± 0.04	4.72 ± 0.30
**PVDF-HFP:PAB (7:3)**	573 ± 4.2	16.12 ± 0.85	1.77 ± 0.13	73.6 ± 6.8	2.95 ± 0.27	0.39 ± 0.03	3.28 ± 0.29
**PVDF-HFP:PAB (6:4)**	562 ± 9.3	14.24 ± 0.40	1.53 ± 0.11	90.3 ± 8.7	3.28 ± 0.53	0.39 ± 0.03	3.11 ± 0.26
**PVDF-HFP:PDES-bAP (9:1)**	685 ± 8.0	2.71 ± 0.08	0.36 ± 0.02	116.2 ± 5.2	3.69 ± 0.64	0.39 ± 0.05	0.73 ± 0.06
**PVDF-HFP:PDES-bAP (8:2)**	848 ± 12.1	2.34 ± 0.16	0.30 ± 0.02	160.5 ± 3.4	4.17 ± 0.87	0.38 ± 0.03	0.61 ± 0.06
**PVDF-HFP:PDES-bAP (7:3)**	703 ± 9.7	0.73 ± 0.09	0.09 ± 0.01	209 ± 7.3	5.37 ± 0.79	0.31 ± 0.02	0.20 ± 0.03
**PVDF-HFP:PDES-bAP (6:4)**	798 ± 4.3	0.02 ± 0.01	0.01 ± 0.01	338.4 ± 25.7	25.66 ± 1.26	0.28 ± 0.03	0.01 ± 0.01
**For size of DSSC 7 × 80 mm^2^**							
**PVDF-HFP:PAB (9:1)**	528 ± 17	6.4 ± 0.51	8.78 ± 0.44	5.49 ± 0.57	0.11 ± 0.06	0.46 ± 0.02	1.57 ± 0.08
